# Sex-related patterns of LDL-C/small dense LDL-C discordance among Japanese adults with non-elevated LDL-C

**DOI:** 10.1016/j.athplu.2026.100616

**Published:** 2026-10-12

**Authors:** Kengo Moriyama

**Affiliations:** Department of Clinical Health Science, Tokai University School of Medicine, Hachioji, Tokyo, Japan

**Keywords:** Small dense LDL cholesterol, LDL-C discordance, Triglycerides, Sex differences, Apolipoprotein B

## Abstract

**Background:**

Low-density lipoprotein cholesterol (LDL-C) remains central to lipid-based cardiovascular risk assessment, but LDL-C alone may not fully capture residual atherogenic lipoprotein burden. Small dense LDL cholesterol (sdLDL-C) is influenced by LDL-C and triglyceride (TG)-rich lipoprotein metabolism, and elevated sdLDL-C may be overlooked when LDL-C is not elevated. Whether LDL-C/sdLDL-C discordance differs by sex across TG strata remains unclear.

**Methods:**

This cross-sectional study included 1879 Japanese adults undergoing annual health check-ups in 2020. The primary analysis included participants with LDL-C <140 mg/dL. Discordance was defined as sdLDL-C ≥35 mg/dL. Discordance prevalence was compared between men and women overall and across TG strata. Apolipoprotein B (ApoB), malondialdehyde-modified LDL (MDA-LDL), high-density lipoprotein 2 cholesterol (HDL2-C), high-density lipoprotein 3 cholesterol (HDL3-C), the HDL2-C/HDL3-C ratio, and high-molecular-weight adiponectin (HMW-adiponectin) were compared by discordance status.

**Results:**

Among participants with LDL-C <140 mg/dL, discordance was more frequent in men than in women [259/917 (28.2%) vs. 42/438 (9.6%), P < 0.001]. This sex difference persisted at TG < 150 mg/dL [16.0% vs. 4.2%, P < 0.001] and TG < 100 mg/dL [7.5% vs. 2.4%, P = 0.0015], but not at TG ≥ 150 mg/dL [74.1% vs. 71.4%, P = 0.835]. Discordant participants had higher ApoB and MDA-LDL and lower HDL2-C, HDL2-C/HDL3-C ratio, and HMW-adiponectin.

**Conclusions:**

LDL-C/sdLDL-C discordance was more frequent in men, particularly in lower TG strata, and was accompanied by an adverse atherogenic lipid profile.

## 1 Background

Low-density lipoprotein cholesterol (LDL-C) is the primary target of lipid management and a central marker in cardiovascular risk assessment. However, LDL particles are heterogeneous in size and density, and qualitative differences in LDL composition may influence atherogenic potential beyond total LDL-C concentration [1], [2]. Moreover, residual cardiovascular risk may persist even when LDL-C is low or not markedly elevated [3].

Small dense LDL (sdLDL) particles have several atherogenic properties, including increased arterial wall penetration and retention, prolonged plasma residence time, and greater susceptibility to oxidative modification [1], [4]. Epidemiological studies have demonstrated that elevated small dense LDL cholesterol (sdLDL-C) levels are associated with incident cardiovascular disease independently of LDL-C [5], [6]. Because sdLDL-C represents cholesterol carried within a subset of LDL particles, individuals with both elevated LDL-C and elevated sdLDL-C may be identified through conventional LDL-C-based screening and treatment strategies. In contrast, individuals with elevated sdLDL-C despite non-elevated LDL-C may be overlooked if risk assessment relies mainly on LDL-C.

Triglyceride-rich lipoprotein metabolism plays a key role in sdLDL formation. Elevated triglyceride (TG) levels promote cholesteryl ester transfer protein-mediated lipid exchange and hepatic lipase-driven hydrolysis, resulting in the formation of smaller and denser LDL particles [2]. Accordingly, elevated sdLDL-C is frequently observed in individuals with hypertriglyceridemia, metabolic syndrome, and insulin-resistant metabolic states. However, evaluating LDL-C/sdLDL-C discordance across TG strata may help clarify whether elevated sdLDL-C is merely a reflection of hypertriglyceridemia or whether discordance is also present among individuals without elevated TG.

In Japan, an LDL-C level of 140 mg/dL is used in screening criteria for elevated LDL-C [7]. Therefore, individuals with LDL-C below this threshold represent a clinically relevant group in whom sdLDL-C elevation may not be apparent from LDL-C-based assessment alone. In this context, LDL-C/sdLDL-C discordance may reflect an apolipoprotein B (ApoB)-rich, TG-related atherogenic phenotype that is not fully captured by LDL-C alone [8].

Sex differences in lipid and lipoprotein metabolism are well recognized. Compared with men, women generally have higher HDL-C and lower TG-rich lipoprotein levels before menopause, whereas lipid profiles may become more atherogenic after menopause [9]. Because LDL-C, HDL-C, and TG levels differ by sex, the relationship between LDL-C and sdLDL-C may also differ between men and women. However, whether LDL-C/sdLDL-C discordance among individuals with non-elevated LDL-C differs by sex, particularly across TG strata, remains unclear.

Although sdLDL-C has been studied extensively in high-risk or disease-specific cohorts, less is known about the clinical and lipid-related characteristics of individuals with elevated sdLDL-C despite non-elevated LDL-C among adults undergoing annual health check-ups. In particular, it remains unclear whether this discordance phenotype is accompanied by adverse advanced lipid-related markers, such as ApoB, oxidatively modified LDL, high-density lipoprotein subfractions, and high-molecular-weight adiponectin.

Therefore, this study aimed to characterize sex-related patterns of LDL-C/sdLDL-C discordance among Japanese adults undergoing annual health check-ups, with particular focus on individuals with LDL-C <140 mg/dL. We further examined whether the prevalence of this discordance differed between men and women across TG strata, and whether discordance was accompanied by an adverse advanced lipid-related marker profile.

## 2 Methods

### 2.1 Study subjects

This cross-sectional study included 1879 adults (1238 men and 641 women) who underwent comprehensive health check-ups at Tokai University Hachioji Hospital in 2020 and had available measurements of LDL-C, sdLDL-C, and related metabolic variables. Eligible participants were adults aged ≥18 years who underwent routine anthropometric measurements, blood pressure assessment, and fasting blood sampling as part of the health check-up program. Participants with hypertension, diabetes, dyslipidemia, or current pharmacological treatment were not excluded. Medical history, medication use, and lifestyle factors were obtained using standardized self-administered questionnaires and confirmed through interviews conducted by trained nurses at the time of the health check-up. Lifestyle factors included current smoking, exercise habits, and alcohol intake. Weekly alcohol intake was calculated from self-reported drinking frequency and amount and expressed as grams per week.

### 2.2 Measurements

Waist circumference was measured at the umbilical level during slight expiration in the standing position. Blood pressure was measured in the seated position on the right upper arm using an automated sphygmomanometer (TM-2655P; A&D, Tokyo, Japan). Blood samples were collected in the morning after an overnight fast. Fasting plasma glucose (FPG) was measured using the hexokinase/glucose-6-phosphate dehydrogenase method (L-type Glu 2; Wako Pure Chemicals, Osaka, Japan). Fasting immunoreactive insulin was measured using a fluorescence enzyme immunoassay (ST AIA-PACK IRI; Tosoh, Tokyo, Japan). Insulin resistance was estimated using the homeostasis model assessment of insulin resistance (HOMA-IR), calculated as FPG (mg/dL) × fasting immunoreactive insulin (μIU/mL)/405 [10]. Serum LDL-C, HDL-C, and TG levels were measured using enzymatic colorimetric methods (Determiner L LDL-C, Determiner L HDL-C, and Determiner L TG II; Kyowa Medex, Tokyo, Japan). sdLDL-C was measured using a homogeneous assay based on a selective detergent method (sdLDL-EX; Denka Seiken, Tokyo, Japan). High-density lipoprotein 2 cholesterol (HDL2-C) and high-density lipoprotein 3 cholesterol (HDL3-C) were measured by ultracentrifugation. High-molecular-weight adiponectin (HMW-adiponectin) was measured using a chemiluminescent enzyme immunoassay. Apolipoprotein B (ApoB) was measured by turbidimetric immunoassay. Malondialdehyde-modified LDL (MDA-LDL) was measured by enzyme-linked immunosorbent assay. High-sensitivity C-reactive protein (hs-CRP) was measured using a latex agglutination method (CRP-HG; Eiken Chemical Co., Ltd., Tokyo, Japan).

### 2.3 Definition of LDL-C/sdLDL-C discordance

For the primary discordance analysis, participants with LDL-C <140 mg/dL were selected because this threshold corresponds to the Japanese screening criterion for elevated LDL-C [7]. LDL-C/sdLDL-C discordance was defined as LDL-C <140 mg/dL and sdLDL-C ≥35 mg/dL. The sdLDL-C threshold of 35 mg/dL was selected based on previous Japanese prospective data showing that this level is associated with increased coronary heart disease risk [6]. Within the LDL-C <140 mg/dL analysis population, participants with sdLDL-C ≥35 mg/dL were classified as discordant, and those with sdLDL-C <35 mg/dL were classified as non-discordant.

### 2.4 TG-stratified analysis

To evaluate whether the prevalence of LDL-C/sdLDL-C discordance differed by sex according to TG status, discordance prevalence was compared between men and women in the overall LDL-C <140 mg/dL analysis population and within TG strata. TG strata included TG < 150 mg/dL, TG < 100 mg/dL, TG 100–149 mg/dL, and TG ≥ 150 mg/dL. The TG < 150 mg/dL category included participants with TG < 100 mg/dL and TG 100–149 mg/dL.

### 2.5 Statistical analysis

Continuous variables are expressed as mean ± standard deviation or median (interquartile range), as appropriate according to their distribution. Categorical variables are presented as numbers (percentages). Comparisons between men and women were performed using Welch's *t*-test for approximately normally distributed continuous variables, the Mann–Whitney *U* test for non-normally distributed continuous variables, and the chi-square test or Fisher's exact test for categorical variables, as appropriate. In the primary LDL-C <140 mg/dL analysis population, baseline characteristics were compared between men and women. The prevalence of LDL-C/sdLDL-C discordance was compared between men and women overall and within TG strata. Odds ratios and 95% confidence intervals were calculated to compare the odds of discordance in men with those in women within each analysis set. P values for sex comparisons of discordance prevalence were calculated using Fisher's exact test.

Advanced lipid-related markers, hs-CRP, and HMW-adiponectin were compared between discordant and non-discordant participants separately in men and women. These variables included ApoB, MDA-LDL, HDL2-C, HDL3-C, the HDL2-C/HDL3-C ratio, hs-CRP, and HMW-adiponectin. Variables are presented as mean ± standard deviation or median (interquartile range), as appropriate, and comparisons were performed using Welch’s t-test or the Mann–Whitney U test, as appropriate. Sensitivity analyses were performed after excluding participants receiving lipid-lowering medication and after excluding those receiving lipid-lowering or diabetes medication. In addition, the primary analysis was repeated using stricter LDL-C thresholds of <130 mg/dL and <115 mg/dL while retaining the sdLDL-C threshold of ≥35 mg/dL. All statistical analyses were performed using Python (version 3.12.12; Python Software Foundation, Wilmington, DE, USA) with the SciPy and statsmodels libraries. A two-sided P value < 0.05 was considered statistically significant.

## 3 Results

### 3.1 Characteristics of participants with LDL-C <140 mg/dL and LDL-C/sdLDL-C distribution

Among the 1879 participants included in the overall study population, 1355 had LDL-C <140 mg/dL and were included in the primary analysis. Of these, 917 were men and 438 were women. In the overall study population, men had higher BMI, waist circumference, blood pressure, FPG, HOMA-IR, TG, and sdLDL-C levels, whereas women had higher LDL-C and HDL-C levels (Supplementary Table 1). The proportion of participants with LDL-C <140 mg/dL was 74.1% in men and 68.3% in women. In the LDL-C <140 mg/dL subgroup, age was almost identical between men and women, and LDL-C levels did not differ significantly. In contrast, men had higher BMI, waist circumference, systolic and diastolic blood pressure, FPG, HOMA-IR, TG, sdLDL-C, and hs-CRP levels, whereas women had higher HDL-C levels (Table 1). Current smoking and alcohol intake differed substantially by sex.

**Table 1:** Characteristics of participants with LDL-C <140 mg/dL.

a. Anthropometric, clinical, and biochemical characteristics			P value
Variable	Men (n = 917)	Women (n = 438)	
Age, years	58.7 ± 12.8	58.7 ± 13.9	0.961
BMI, kg/m^2^	24.2 ± 3.2	22.3 ± 3.7	<0.001
WC, cm	86.0 ± 8.8	80.5 ± 10.5	<0.001
SBP, mmHg	127.7 ± 17.3	123.4 ± 19.5	<0.001
DBP, mmHg	80.1 ± 12.4	73.4 ± 12.8	<0.001
LDL-C, mg/dL	109.5 ± 19.7	110.0 ± 18.9	0.631
HDL-C, mg/dL	60.7 ± 15.6	76.4 ± 18.0	<0.001
FPG, mg/dL	99.0 (93.0, 108.0)	94.0 (88.0, 102.8)	<0.001
HbA1c(NGSP), %	5.6 (5.4, 5.9)	5.6 (5.4, 5.8)	0.874
HOMA-IR	1.3 (0.9, 2.1)	1.1 (0.8, 1.8)	<0.001
TG, mg/dL	94.0 (67.0, 138.0)	73.5 (55.0, 98.0)	<0.001
hs-CRP, mg/dL	0.05 (0.03, 0.10)	0.04 (0.02, 0.09)	0.005
sdLDL-C, mg/dL	27.2 (20.9, 36.5)	22.4 (18.3, 28.0)	<0.001
Discordance, n (%)	259 (28.2)	42 (9.6)	<0.001

b. Lifestyle characteristics			

Current smoking, n (%)	191 (20.8)	37 (8.4)	<0.001
Current exercise, n (%)	337 (36.8)	142 (32.4)	0.119
Alcohol intake category, n (%)			<0.001
Non-drinker	368 (40.1)	323 (73.7)	
Low	209 (22.8)	59 (13.5)	
Moderate	154 (16.8)	28 (6.4)	
High	186 (20.3)	28 (6.4)	

Discordance was defined as sdLDL-C ≥35 mg/dL among participants with LDL-C <140 mg/dL. Alcohol categories: non-drinker = 0 g/week; low = 1 to 139 g/week; moderate = 140 to 279 g/week; high = ≥280 g/week. Variables are expressed as mean ± standard deviation, median (interquartile range), or n (%). BMI, body mass index; WC, waist circumference; SBP, systolic blood pressure; DBP, diastolic blood pressure; LDL-C, low-density lipoprotein cholesterol; HDL-C, high-density lipoprotein cholesterol; FPG, fasting plasma glucose; HOMA-IR, homeostasis model assessment of insulin resistance; TG, triglycerides; hs-CRP, high-sensitivity C-reactive protein; sdLDL-C, small dense low-density lipoprotein cholesterol.

P values were calculated using Welch's *t*-test, Mann–Whitney *U* test, chi-square test, or Fisher's exact test, as appropriate.

The sex-specific distribution of LDL-C and sdLDL-C is shown in Fig. 1. LDL-C/sdLDL-C discordance was observed in both sexes, but it was more frequent in men than in women among participants with LDL-C <140 mg/dL [259/917 (28.2%) vs. 42/438 (9.6%), P < 0.001]. Thus, although LDL-C levels were comparable between sexes in the primary analysis population, the proportion of participants with elevated sdLDL-C differed markedly by sex.

**Fig. 1:**
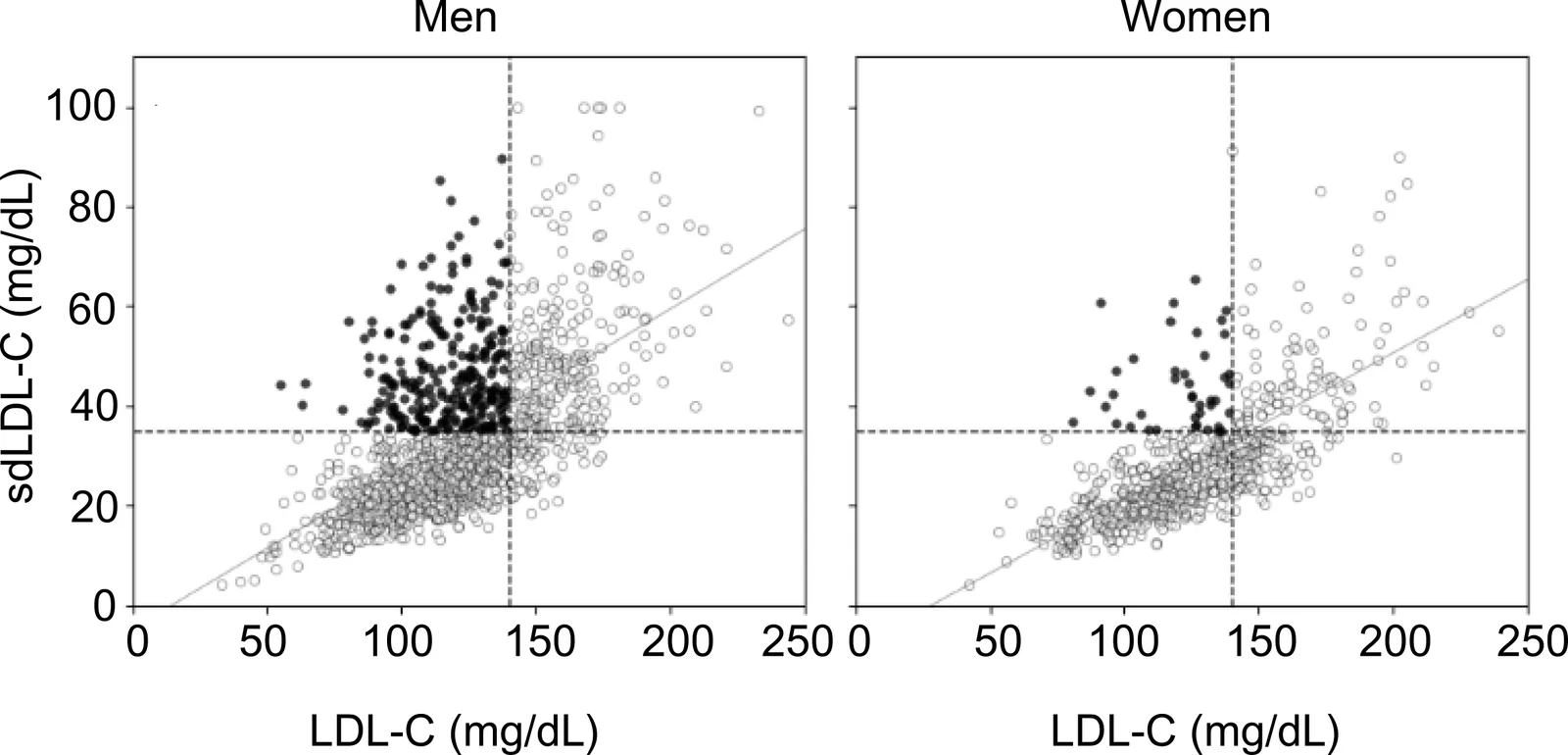
Sex-stratified distribution of LDL-C and sdLDL-C in the study population. Scatter plots show the relationship between LDL-C and sdLDL-C in men and women. The vertical line indicates the LDL-C threshold of 140 mg/dL, and the horizontal line indicates the sdLDL-C threshold of 35 mg/dL. LDL-C/sdLDL-C discordance was defined as sdLDL-C ≥35 mg/dL among participants with LDL-C <140 mg/dL. LDL-C, low-density lipoprotein cholesterol; sdLDL-C, small dense low-density lipoprotein cholesterol.

### 3.2 Sex-stratified prevalence of LDL-C/sdLDL-C discordance across triglyceride strata

The prevalence of LDL-C/sdLDL-C discordance differed markedly by sex across TG strata (Fig. 2 and Supplementary Table 2). In the overall LDL-C <140 mg/dL analysis population, men had higher odds of discordance than women [odds ratio (OR) 3.71, 95% confidence interval (CI) 2.62–5.26]. Among participants with TG < 150 mg/dL, discordance was more frequent in men than in women [116/724 (16.0%) vs. 17/403 (4.2%), OR 4.33, 95% CI 2.56–7.32, P < 0.001]. This sex difference persisted even among participants with TG < 100 mg/dL [37/491 (7.5%) vs. 8/331 (2.4%), OR 3.29, 95% CI 1.51–7.16, P = 0.0015], although the number of discordant women in this stratum was small. In contrast, among participants with TG ≥ 150 mg/dL, discordance was highly prevalent in both sexes, with no significant difference between men and women [143/193 (74.1%) vs. 25/35 (71.4%), OR 1.14, 95% CI 0.51–2.55, P = 0.835]. These findings suggest that discordance was common in the presence of elevated TG in both sexes, whereas discordance persisted more frequently in men than in women in lower TG strata.

**Fig. 2:**
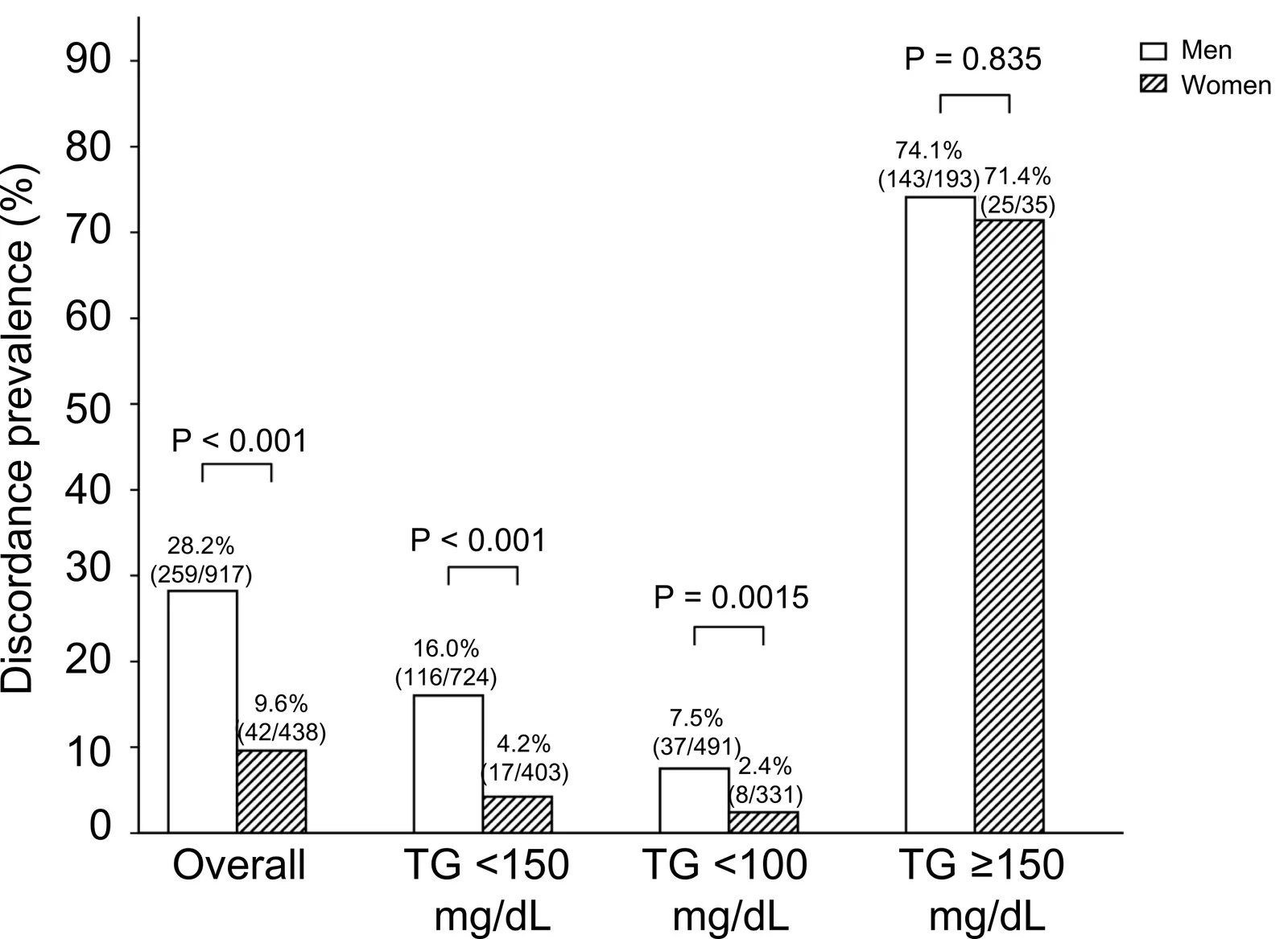
TG-stratified prevalence of LDL-C/sdLDL-C discordance by sex. Bars indicate the proportion of discordant participants among those with LDL-C <140 mg/dL in each TG category. Discordance was defined as sdLDL-C ≥35 mg/dL among participants with LDL-C <140 mg/dL. Values indicate the number and percentage of discordant participants in each group. P values were calculated using Fisher's exact test. LDL-C, low-density lipoprotein cholesterol; TG, triglycerides; sdLDL-C, small dense low-density lipoprotein cholesterol.

Sensitivity analyses excluding participants receiving lipid-lowering medication and those receiving lipid-lowering or diabetes medication showed similar sex-related patterns of LDL-C/sdLDL-C discordance. Discordance remained more frequent in men than in women overall and in lower TG strata, whereas no significant sex difference was observed among participants with TG ≥ 150 mg/dL (Supplementary Table 3). Similar findings were observed when a stricter LDL-C threshold of <130 mg/dL was used to define the non-elevated LDL-C population (Supplementary Table 4). Similar sex-related patterns were also observed when an LDL-C threshold of <115 mg/dL was used, although the comparison in the TG < 100 mg/dL stratum was limited by the small number of discordant women (Supplementary Table 5).

### 3.3 Advanced lipid-related markers and HMW-adiponectin according to LDL-C/sdLDL-C discordance

Advanced lipid-related markers and HMW-adiponectin according to discordance status are shown in Table 2. In men, discordant participants had higher ApoB levels (98.4 ± 11.2 vs. 80.6 ± 13.4 mg/dL, P < 0.001) and higher median MDA-LDL levels [126.0 (108.5, 152.0) vs. 96.0 (76.0, 115.0) U/L, P < 0.001] than non-discordant participants. Similar differences were observed in women, with higher ApoB levels (99.5 ± 12.0 vs. 79.6 ± 12.7 mg/dL, P < 0.001) and higher median MDA-LDL levels [107.5 (93.0, 133.0) vs. 84.0 (68.0, 102.2) U/L, P < 0.001] in discordant participants. In both sexes, discordant participants had lower HDL2-C, HDL2-C/HDL3-C ratio, and HMW-adiponectin levels than non-discordant participants. In contrast, HDL3-C did not differ significantly according to discordance status in either men or women. hs-CRP levels did not differ significantly according to discordance status in men, whereas they were higher in discordant than in non-discordant women. These findings indicate that LDL-C/sdLDL-C discordance was associated with higher atherogenic particle-related markers and an unfavorable HDL subfraction and adiponectin profile in both sexes.

**Table 2:** Characteristics according to LDL-C/sdLDL-C discordance among participants with LDL-C <140 mg/dL.

Variable	Men			Women		
	Non-discordance (n = 658)	Discordance (n = 259)	P value	Non-discordance (n = 396)	Discordance (n = 42)	P value
Age, years	59.0 ± 13.3	57.9 ± 11.5	0.218	58.4 ± 14.1	61.5 ± 11.7	0.105
BMI, kg/m^2^	23.9 ± 3.2	25.1 ± 3.1	<0.001	22.1 ± 3.6	24.1 ± 4.5	0.008
WC, cm	85.0 ± 9.0	88.4 ± 8.0	<0.001	79.9 ± 10.2	85.7 ± 11.6	0.003
SBP, mmHg	125.7 ± 17.2	132.7 ± 16.7	<0.001	122.8 ± 19.4	129.1 ± 18.9	0.045
DBP, mmHg	78.7 ± 12.3	83.6 ± 12.1	<0.001	73.0 ± 12.9	78.0 ± 11.2	0.009
LDL-C, mg/dL	106.5 ± 20.3	117.0 ± 15.9	<0.001	108.9 ± 18.9	120.5 ± 16.3	<0.001
HDL-C, mg/dL	63.0 ± 14.9	54.7 ± 15.6	<0.001	77.8 ± 17.6	63.4 ± 16.5	<0.001
FPG, mg/dL	98.0 (92.0, 106.0)	102.0 (96.0, 113.0)	<0.001	94.0 (88.0, 102.0)	98.0 (91.0, 107.0)	0.065
HbA1c(NGSP), %	5.6 (5.3, 5.9)	5.6 (5.4, 5.9)	0.063	5.6 (5.4, 5.8)	5.6 (5.4, 6.0)	0.383
HOMA-IR	1.2 (0.8, 1.9)	1.7 (1.1, 2.4)	<0.001	1.1 (0.8, 1.7)	1.7 (1.0, 3.3)	<0.001
TG, mg/dL	80.0 (60.2, 106.0)	158.0 (118.0, 231.0)	<0.001	69.0 (52.8, 92.0)	157.5 (128.5, 192.2)	<0.001
hs-CRP, mg/dL	0.05 (0.02, 0.10)	0.05 (0.03, 0.10)	0.083	0.04 (0.02, 0.09)	0.06 (0.03, 0.16)	0.018
sdLDL-C, mg/dL	23.4 (19.4, 28.4)	43.9 (39.0, 53.2)	<0.001	21.9 (18.0, 26.1)	42.0 (37.0, 47.1)	<0.001
ApoB, mg/dL	80.6 ± 13.4	98.4 ± 11.2	<0.001	79.6 ± 12.7	99.5 ± 12.0	<0.001
MDA-LDL, U/L	96.0 (76.0, 115.0)	126.0 (108.5, 152.0)	<0.001	84.0 (68.0, 102.2)	107.5 (93.0, 133.0)	<0.001
HDL2-C, mg/dL	37.5 ± 12.6	30.6 ± 12.9	<0.001	51.1 ± 16.2	38.9 ± 15.1	<0.001
HDL3-C, mg/dL	22.0 ± 3.7	22.2 ± 4.3	0.613	21.5 ± 3.9	21.6 ± 4.3	0.882
HDL2-C/HDL3-C ratio	1.6 (1.3, 2.0)	1.3 (1.1, 1.5)	<0.001	2.2 (1.8, 2.9)	1.6 (1.3, 2.2)	<0.001
HMW-adiponectin, μg/mL	2.8 (1.8, 4.1)	2.0 (1.4, 3.0)	<0.001	5.2 (3.5, 7.9)	3.9 (2.2, 5.7)	<0.001

Values are presented as mean ± SD or median (interquartile range). Discordance was defined as sdLDL-C ≥35 mg/dL among participants with LDL-C <140 mg/dL. P values were calculated using Welch's *t*-test or the Mann–Whitney *U* test, as appropriate.

BMI, body mass index; WC, waist circumference; SBP, systolic blood pressure; DBP, diastolic blood pressure; LDL-C, low-density lipoprotein cholesterol; HDL-C, high-density lipoprotein cholesterol; FPG, fasting plasma glucose; HOMA-IR, homeostasis model assessment of insulin resistance; TG, triglycerides; hs-CRP, high-sensitivity C-reactive protein; sdLDL-C, small dense low-density lipoprotein cholesterol; ApoB, apolipoprotein B; MDA-LDL, malondialdehyde-modified LDL; HDL2-C, high-density lipoprotein 2 cholesterol; HDL3-C, high-density lipoprotein 3 cholesterol; HMW-adiponectin, high-molecular-weight adiponectin.

## 4 Discussion

In this cross-sectional study of Japanese adults undergoing health check-ups, LDL-C/sdLDL-C discordance was observed among participants with non-elevated LDL-C and was substantially more frequent in men than in women. This sex difference persisted after restriction to participants with TG < 150 mg/dL and even to those with TG < 100 mg/dL, whereas discordance was highly prevalent in both sexes among participants with TG ≥ 150 mg/dL. In addition, discordant participants had higher ApoB and MDA-LDL levels and lower HDL2-C, HDL2-C/HDL3-C ratio, and HMW-adiponectin levels in both sexes. These findings suggest that LDL-C/sdLDL-C discordance identifies an adverse atherogenic lipid phenotype that is not captured by LDL-C alone, with a sex-related difference in its prevalence and TG-stratified distribution.

LDL-C remains the principal lipid marker used in cardiovascular risk assessment and lipid-lowering treatment decisions. However, LDL-C concentration does not necessarily reflect the number, size, or oxidative modification of atherogenic lipoprotein particles. Previous Japanese cohort data have shown that elevated sdLDL-C is associated with incident coronary heart disease, supporting its clinical relevance beyond conventional LDL-C assessment [6]. In addition, TG-rich lipoprotein cholesterol and sdLDL-C have been shown to be associated with incident cardiovascular disease, suggesting that these lipoprotein fractions contribute to atherogenesis beyond conventional LDL-C measurement [11]. In the present study, participants with LDL-C <140 mg/dL but elevated sdLDL-C showed higher ApoB and MDA-LDL levels than non-discordant participants. This finding is clinically relevant because ApoB reflects the burden of atherogenic lipoprotein particles and may provide information beyond LDL-C in settings where LDL-C and particle burden are discordant [8], while MDA-LDL represents an oxidatively modified LDL fraction. Thus, discordance between LDL-C and sdLDL-C may represent a phenotype in which LDL-C appears acceptable, but the underlying atherogenic particle burden remains unfavorable. Recent studies have examined sdLDL-C discordance in relation to cardiometabolic and subclinical atherosclerotic outcomes. In Chinese cohort studies, discordance between sdLDL-C and LDL-C has been associated with triglyceride-related cardiovascular risk and incident hypertension [12], [13], and discordantly high sdLDL-C relative to LDL-C or non-HDL-C has also been associated with carotid plaque [14]. These findings support the concept that sdLDL-C may capture lipid-related and atherosclerotic risk not fully reflected by conventional cholesterol measures. However, these studies were conducted primarily in Chinese populations and focused mainly on cardiovascular, hypertension, or carotid plaque outcomes. In contrast, the present study focused on Japanese adults with LDL-C below the Japanese screening threshold for elevated LDL-C and characterized the sex- and TG-stratified distribution of LDL-C/sdLDL-C discordance together with ApoB, MDA-LDL, HDL subfractions, HMW-adiponectin, and hs-CRP. To our knowledge, few studies have examined this specific discordance phenotype in relation to sex, TG strata, and advanced lipid-related markers in an annual health check-up setting.

A notable finding of this study was the marked sex difference in the prevalence of LDL-C/sdLDL-C discordance. Among participants with LDL-C <140 mg/dL, discordance was approximately three times more frequent in men than in women. This difference cannot be explained by LDL-C levels alone, because LDL-C was similar between men and women in the primary analysis population. Instead, men had higher TG, sdLDL-C, BMI, waist circumference, FPG, and blood pressure, and lower HDL-C, suggesting a more insulin-resistant and TG-rich metabolic background. These characteristics are compatible with the known metabolic pathway in which insulin resistance and TG-rich lipoprotein accumulation promote lipid exchange and lipolysis, resulting in the formation of smaller and denser LDL particles [15].

The TG-stratified analysis further clarified this sex-related pattern. Among participants with TG ≥ 150 mg/dL, discordance was highly prevalent in both men and women, indicating that hypertriglyceridemia is strongly associated with sdLDL-C elevation regardless of sex. In contrast, among participants with TG < 150 mg/dL and <100 mg/dL, discordance remained more frequent in men than in women. Sex differences in lipid and lipoprotein metabolism are well recognized, with premenopausal women generally showing higher HDL-C and lower TG-rich lipoprotein levels than men [8], and these factors may partly contribute to sex-related differences in sdLDL-C distribution. However, because menopausal status was unavailable and the number of discordant women was limited, the present findings should be interpreted as sex-stratified descriptive patterns rather than evidence of sex-specific mechanisms. These findings may be partly explained by TG-driven lipoprotein remodeling. In the presence of elevated TG-rich lipoproteins, cholesteryl ester transfer protein promotes lipid exchange between TG-rich lipoproteins, LDL, and HDL particles. Subsequent hydrolysis by hepatic lipase can generate smaller and denser LDL particles and may reduce larger HDL2 particles, thereby lowering HDL2-C levels and the HDL2-C/HDL3-C ratio. This mechanism is compatible with the higher sdLDL-C levels and lower HDL2-C and HDL2-C/HDL3-C ratio observed in discordant participants in both sexes. In addition, estrogens influence lipid and lipoprotein metabolism, including HDL-C and TG-rich lipoprotein metabolism, and may partly contribute to the lower prevalence of LDL-C/sdLDL-C discordance in women, particularly in lower TG strata. However, because menopausal status, hormone therapy, and sex hormone levels were not available, this interpretation remains speculative.

The advanced lipid-related marker profile provides additional insight into the clinical meaning of discordance. In both sexes, discordant participants had higher ApoB and MDA-LDL levels, lower HDL2-C and HDL2-C/HDL3-C ratio, and lower HMW-adiponectin levels. In contrast, HDL3-C did not differ significantly by discordance status. These findings suggest that LDL-C/sdLDL-C discordance is not merely an isolated elevation of sdLDL-C but rather reflects a broader atherogenic and metabolically unfavorable phenotype. The lower HDL2-C/HDL3-C ratio and HMW-adiponectin levels in discordant participants are consistent with an insulin-resistant background, as the HDL2-C/HDL3-C ratio has been associated with insulin resistance, HMW-adiponectin, and metabolic syndrome components in Japanese individuals [16], and HMW-adiponectin has been reported to be useful for predicting insulin resistance and metabolic syndrome [17].

The present findings may have implications for risk assessment in health check-up settings. In routine practice, individuals with LDL-C below the conventional abnormal threshold may be considered to have relatively acceptable LDL-related risk. However, our results show that a subset of such individuals, particularly men and those with elevated TG, have elevated sdLDL-C and an adverse atherogenic lipid profile. Therefore, sdLDL-C measurement may help identify residual lipid-related risk that is not apparent from LDL-C alone. Importantly, our findings do not imply that sdLDL-C should be measured universally, but rather suggest that sex and triglyceride status may help identify individuals in whom sdLDL-C assessment is more informative.

Several limitations should be acknowledged. First, this was a cross-sectional study, and causal relationships between triglyceride metabolism, sdLDL-C elevation, and atherogenic marker profiles cannot be inferred. Second, cardiovascular outcomes were not available; therefore, the prognostic significance of LDL-C/sdLDL-C discordance could not be directly evaluated. Third, the number of women with discordance was relatively small, especially in the lower TG strata, which limits the strength of sex-specific conclusions. Fourth, menopausal status, hormone therapy, and sex hormone levels were not available. Because the mean age of women in this study was close to the postmenopausal age range, menopausal status may have influenced lipid metabolism and sdLDL-C distribution. Fifth, this was a single-center study of Japanese health check-up participants, and the findings may not be generalizable to other ethnic groups or clinical populations. Finally, although several advanced lipid-related markers were evaluated, other potentially relevant markers, such as lipoprotein(a) or LDL particle number, were not included.

In conclusion, LDL-C/sdLDL-C discordance among individuals with non-elevated LDL-C was substantially more frequent in men than in women, particularly in lower TG strata, whereas elevated TG was associated with a high prevalence of discordance in both sexes. Discordant participants showed higher ApoB and MDA-LDL levels and lower HDL2-C, HDL2-C/HDL3-C ratio, and HMW-adiponectin levels, indicating an adverse atherogenic lipid phenotype. These findings suggest that sdLDL-C assessment may provide additional information for detecting residual lipid-related risk not captured by LDL-C alone, especially when interpreted in relation to sex and TG status.

## Ethics declaration

The authors have NOT obtained informed consent from participants or their legal representatives. Written and verbal informed consent were not obtained because this study retrospectively used existing health check-up data. The requirement for individual informed consent was waived by the Ethics Committee of Tokai University School of Medicine (protocol no. 20R-279). Information about the study was made publicly available, and participants were given the opportunity to opt out of the use of their data.

This study was performed in compliance with relevant laws, regulatory frameworks and guidelines where the research took place. This study was approved by the Ethics Committee of Tokai University School of Medicine. (Approval No. 20R-279)

## Data availability

The datasets generated and/or analyzed during the current study are not publicly available due to Tokai University regulations but are available from the corresponding author upon reasonable request.

## Declaration of interest statement

The authors declare that they have no known competing financial interests or personal relationships that could have appeared to influence the work reported in this paper.
