# Supplementary material for: Sex-related patterns of LDL-C/small dense LDL-C discordance among Japanese adults with non-elevated LDL-C
Source: Atheroscler Plus. 2026 Oct 12;66:100616. doi: 10.1016/j.athplu.2026.100616 (PMC13721921; doi:10.1016/j.athplu.2026.100616)
Supplement: Supplementary file 1 [file mmc1.docx]

Supplementary Table 1. Characteristics of the overall study population

| a. Anthropometric, clinical, and biochemical characteristics | | |  |  |
| --- | --- | --- | --- | --- |
| Variable | Men (n=1238) | Women (n=641) | P value |  |
| Age, years | 58.2 ± 12.7 | 59.6 ± 13.0 | 0.031 |  |
| BMI, kg/m² | 24.3 ± 3.2 | 22.6 ± 3.7 | <0.001 |  |
| WC, cm | 86.2 ± 8.8 | 81.3 ± 10.3 | <0.001 |  |
| SBP, mmHg | 128.2 ± 17.4 | 125.1 ± 20.1 | <0.001 |  |
| DBP, mmHg | 80.4 ± 12.4 | 74.1 ± 13.0 | <0.001 |  |
| LDL-C, mg/dL | 122.6 ± 29.5 | 126.4 ± 30.9 | 0.011 |  |
| HDL-C, mg/dL | 59.9 ± 15.3 | 75.1 ± 17.7 | <0.001 |  |
| FPG, mg/dL | 99.0 (93.0, 107.0) | 94.0 (89.0, 102.0) | <0.001 |  |
| HbA1c(NGSP), % | 5.6 (5.4, 5.9) | 5.6 (5.4, 5.8) | 0.333 |  |
| HOMA-IR | 1.4 (0.9, 2.1) | 1.2 (0.8, 1.9) | <0.001 |  |
| TG, mg/dL | 101.0 (72.0, 147.0) | 79.0 (57.0, 112.0) | <0.001 |  |
| hs-CRP, mg/dL | 0.05 (0.03, 0.10) | 0.04 (0.02, 0.10) | 0.003 |  |
| sdLDL-C, mg/dL | 31.4 (22.8, 43.1) | 26.0 (20.5, 33.7) | <0.001 |  |
| LDL-C <140 mg/dL, n (%) | 917 (74.1) | 438 (68.3) | 0.01 |  |
|  |  |  |  |  |
| b. Lifestyle characteristics |  |  |  |  |
| Current smoking, n (%) | 265 (21.4) | 51 (8.0) | <0.001 | |
| Current exercise, n (%) | 440 (35.5) | 202 (31.5) | 0.09 |  |
| Alcohol intake category, n (%) |  |  | <0.001 |  |
| Non-drinker | 531 (42.9) | 486 (75.8) |  |  |
| Low | 270 (21.8) | 86 (13.4) |  |  |
| Moderate | 189 (15.3) | 36 (5.6) |  |  |
| High | 248 (20.0) | 33 (5.1) |  |  |
| Discordance was defined as sdLDL-C ≥35 mg/dL among participants with LDL-C <140 mg/dL. Alcohol categories: non-drinker = 0 g/week; low = 1 to 139 g/week; moderate = 140 to 279 g/week; high = ≥280 g/week. Variables are expressed as mean ± standard deviation or median (interquartile range).  BMI, body mass index; SBP, systolic blood pressure; DBP, diastolic blood pressure; LDL-C, low-density lipoprotein cholesterol; HDL-C, high-density lipoprotein cholesterol; FPG, fasting plasma glucose; HOMA-IR, homeostasis model assessment of insulin resistance; TG, triglycerides; hs-CRP, high-sensitivity C-reactive protein; sdLDL-C, small dense low-density lipoprotein cholesterol.  P values were calculated using Welch's t-test, Mann–Whitney U test, or chi-square test, as appropriate. | | | |  |

Supplementary Table 2. TG-stratified prevalence of LDL-C/sdLDL-C discordance by sex among participants with LDL-C <140 mg/dL

| Analysis set | Men, discordant n/N (%) | Women, discordant n/N (%) | Odds ratio (95% CI) | P value |
| --- | --- | --- | --- | --- |
| Overall | 259/917 (28.2) | 42/438 (9.6) | 3.71 (2.62–5.26) | <0.001 |
| TG <150 mg/dL | 116/724 (16.0) | 17/403 (4.2) | 4.33 (2.56–7.32) | <0.001 |
| TG <100 mg/dL | 37/491 (7.5) | 8/331 (2.4) | 3.29 (1.51–7.16) | 0.0015 |
| TG 100–149 mg/dL | 79/233 (33.9) | 9/72 (12.5) | 3.59 (1.70–7.60) | <0.001 |
| TG ≥150 mg/dL | 143/193 (74.1) | 25/35 (71.4) | 1.14 (0.51–2.55) | 0.835 |
| Values are presented as n/N (%). The analysis was restricted to participants with LDL-C <140 mg/dL; within this population, discordant participants were defined as those with sdLDL-C ≥35 mg/dL. Odds ratios compare men with women within each TG category. P values were calculated using Fisher’s exact test. The TG <150 mg/dL category includes participants with TG <100 mg/dL and TG 100–149 mg/dL. | | | | |

Supplementary Table 3. Medication-exclusion sensitivity analyses of TG-stratified LDL-C/sdLDL-C discordance prevalence

| a. Excluding participants receiving lipid-lowering medication | | | | |
| --- | --- | --- | --- | --- |
| Analysis set | Men, discordant n/N (%) | Women, discordant n/N (%) | Odds ratio (95% CI) | P value |
| Overall | 183/679 (27.0) | 24/323 (7.4) | 4.60 (2.93–7.20) | <0.001 |
| TG <150 mg/dL | 87/549 (15.8) | 9/302 (3.0) | 6.13 (3.04–12.37) | <0.001 |
| TG <100 mg/dL | 31/384 (8.1) | 5/262 (1.9) | 4.51 (1.73–11.77) | <0.001 |
| TG 100–149 mg/dL | 56/165 (33.9) | 4/40 (10.0) | 4.62 (1.57–13.64) | 0.002 |
| TG ≥150 mg/dL | 96/130 (73.8) | 15/21 (71.4) | 1.13 (0.41–3.15) | 0.794 |
|  |  |  |  |  |
| b. Excluding participants receiving lipid-lowering or diabetes medication | | | | |
| Analysis set | Men, discordant n/N (%) | Women, discordant n/N (%) | Odds ratio (95% CI) | P value |
| Overall | 175/639 (27.4) | 23/317 (7.3) | 4.82 (3.05–7.63) | <0.001 |
| TG <150 mg/dL | 80/513 (15.6) | 8/296 (2.7) | 6.65 (3.17–13.97) | <0.001 |
| TG <100 mg/dL | 26/358 (7.3) | 4/257 (1.6) | 4.95 (1.71–14.37) | <0.001 |
| TG 100–149 mg/dL | 54/155 (34.8) | 4/39 (10.3) | 4.68 (1.58–13.86) | 0.0029 |
| TG ≥150 mg/dL | 95/126 (75.4) | 15/21 (71.4) | 1.23 (0.44–3.43) | 0.787 |
| Values are presented as n/N (%). The analysis was restricted to participants with LDL-C <140 mg/dL; within this population, discordant participants were defined as those with sdLDL-C ≥35 mg/dL. Odds ratios compare men with women within each TG category. P values were calculated using Fisher’s exact test. The TG <150 mg/dL category includes participants with TG <100 mg/dL and TG 100–149 mg/dL. LDL-C, low-density lipoprotein cholesterol; sdLDL-C, small dense low-density lipoprotein cholesterol; TG, triglycerides. | | | | |

Supplementary Table 4. Sensitivity analysis using LDL-C <130 mg/dL as the non-elevated LDL-C threshold

| Analysis set | Men, discordant n/N (%) | Women, discordant n/N (%) | Odds ratio (95% CI) | P value |
| --- | --- | --- | --- | --- |
| Overall | 192/764 (25.1) | 27/365 (7.4) | 4.20 (2.75–6.43) | <0.001 |
| TG <150 mg/dL | 81/608 (13.3) | 10/340 (2.9) | 5.07 (2.59–9.92) | <0.001 |
| TG <100 mg/dL | 24/423 (5.7) | 5/286 (1.7) | 3.38 (1.27–8.97) | 0.011 |
| TG 100–149 mg/dL | 57/185 (30.8) | 5/54 (9.3) | 4.36 (1.65–11.53) | 0.0013 |
| TG ≥150 mg/dL | 111/156 (71.2) | 17/25 (68.0) | 1.16 (0.47–2.88) | 0.814 |
| Values are presented as n/N (%). The analysis was restricted to participants with LDL-C <130 mg/dL; within this population, discordant participants were defined as those with sdLDL-C ≥35 mg/dL. Odds ratios compare men with women within each TG category. P values were calculated using Fisher’s exact test. The TG <150 mg/dL category includes participants with TG <100 mg/dL and TG 100–149 mg/dL. LDL-C, low-density lipoprotein cholesterol; sdLDL-C, small dense low-density lipoprotein cholesterol; TG, triglycerides. | | | | |

Supplementary Table 5. Sensitivity analysis using LDL-C <115 mg/dL as the non-elevated LDL-C threshold

| Analysis set | Men, discordant n/N (%) | Women, discordant n/N (%) | Odds ratio (95% CI) | P value |
| --- | --- | --- | --- | --- |
| Overall | 103/499 (20.6) | 12/235 (5.1) | 4.83 (2.60–8.98) | <0.001 |
| TG <150 mg/dL | 34/396 (8.6) | 4/222 (1.8) | 5.12 (1.79–14.62) | <0.001 |
| TG <100 mg/dL | 12/290 (4.1) | 2/184 (1.1) | 3.93 (0.87–17.76) | 0.091 |
| TG 100–149 mg/dL | 22/106 (20.8) | 2/38 (5.3) | 4.71 (1.05–21.11) | 0.040 |
| TG ≥150 mg/dL | 69/103 (67.0) | 8/13 (61.5) | 1.27 (0.39–4.17) | 0.759 |
| Values are presented as n/N (%). The analysis was restricted to participants with LDL-C <115 mg/dL; within this population, discordant participants were defined as those with sdLDL-C ≥35 mg/dL. Odds ratios compare men with women within each TG category. P values were calculated using Fisher’s exact test. The TG <150 mg/dL category includes participants with TG <100 mg/dL and TG 100–149 mg/dL. LDL-C, low-density lipoprotein cholesterol; sdLDL-C, small dense low-density lipoprotein cholesterol; TG, triglycerides. | | | | |
